# Two simple and rapid methods based on maximum diameter accurately estimate large lesion volumes in acute stroke

**DOI:** 10.1002/brb3.1828

**Published:** 2020-09-09

**Authors:** Anna Kufner, Jonas Stief, Bob Siegerink, Christian Nolte, Matthias Endres, Jochen B. Fiebach

**Affiliations:** ^1^ Department of Neurology Center for Stroke Research Berlin (CSB) Charité – Universitätsmedizin Berlin Germany; ^2^ Berlin Institute of Health (BIH) Berlin Germany; ^3^ Department of Radiology Charité – Universitätsmedizin Berlin Germany

**Keywords:** diffusion‐weighted imaging, magnetic resonance imaging, stroke

## Abstract

**Background:**

We compared two simple and rapid diameter‐based methods (ABC/2, od‐value) in terms of their accuracy in predicting lesion volume >70 ml and >100 ml.

**Methods:**

In 238 DWI images of ischemic stroke patients from the AXIS2 trial, maximum lesion diameter and corresponding maximum orthogonal diameter were measured. Estimation of infarct volume based on od‐value and ABC/2 calculation was compared to volumetric assessments.

**Results:**

Accuracy of od‐value and ABC/2 was similar for >70 ml (92.0 vs. 87.4) and >100 ml (92.9 vs. 93.3). ABC/2 overestimated lesion volume by 29.9%, resulting in a lower specificity.

**Conclusions:**

Od‐value is a robust tool for patient selection in trials.

## Graphical abstract

Assessment of lesion volume of infarcted tissue is often used in stroke trials to select patients most likely to benefit from thrombolysis. Here, we demonstrate that two simple diameter‐based methods can accurately estimate lesion size with high sensitivity and specificity in the time‐sensitive setting of acute stroke.

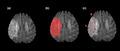

## 1 INTRODUCTION

Large initial lesion volume is an important prognostic imaging biomarker in acute ischemic stroke and, hence, is often used as an exclusion criterion for enrollment in trials (Albers et al., 2006; Lansberg et al., 2007, 2012). Large infarction is associated with worse functional recovery and increased risk of hemorrhagic transformation following thrombolysis (Lansberg et al., 2007). Therefore, infarcts extending >1/3 of middle cerebral artery territory or measuring >100 ml have been proposed as exclusion criteria for treatment (Albers et al., 2006). For endovascular treatment, 70 ml were considered exclusionary (DEFUSE2) (Lansberg et al., 2012). Currently, the gold standard for measuring lesion size is manual delineation of infarct borders on all diffusion‐weighted imaging (DWI) slices. However, this method is time‐consuming and consequently not suitable for the time‐sensitive setting of acute stroke. While visual estimation of infarct volume is often used, it has been shown to be highly variable among readers (Fiebach et al., 2015). Therefore, there is an important need for a faster and more accurate method for estimating lesion volume in the acute setting.

Two alternative methods for the estimation of lesion volume have been proposed in which simple two‐diameter measurements are applied: (1) the ABC/2 method calculated by two maximal orthogonal diameters multiplied by slice thickness (Sims et al., 2009) and (2) the od‐value method calculating only two maximal orthogonal diameters (Fiebach et al., 2015). While Sims and coworkers found ABC/2 to be accurate and reproducible in estimating lesion size (Sims et al., 2009), others criticized its accuracy showing overestimation of volume of up to 63% (Pedraza et al., 2012). A recent study introduced the use of orthogonal DWI diameters (od‐value) and compared this method to visual estimation and ABC/2 in 108 patients (Fiebach et al., 2015). They found that use of specific od‐value cutoffs for 100 ml and 70 ml infarction was more accurate than visual estimation and ABC/2.

As of yet, all proposed diameter‐based methods have only been analyzed in relatively small cohorts of patients from monocentric studies (Fiebach et al., 2015; Pedraza et al., 2012; Sims et al., 2009). Therefore, due to the high clinical relevancy of this topic, we compared the two abovementioned methods in a large, multicenter database of patients for the prediction of large lesion volume.

## 2 METHODS

### 2.1 Patient cohort

This is a post hoc analysis of the AXIS2 trial (NCT00927836), in which consecutive ischemic stroke patients were screened within 9 hr of symptom‐onset with standardized stroke magnetic resonance imaging protocol (Ringelstein et al., 2013).

### 2.2 Ethics statement

The AXIS2 trial was approved by all local ethics committees of local recruiting sites. All patients provided written informed consent; design and content of the consent form were according to country regulations and proved by the lead and local ethics committees.

### 2.3 Image analysis

**FIGURE 1:**
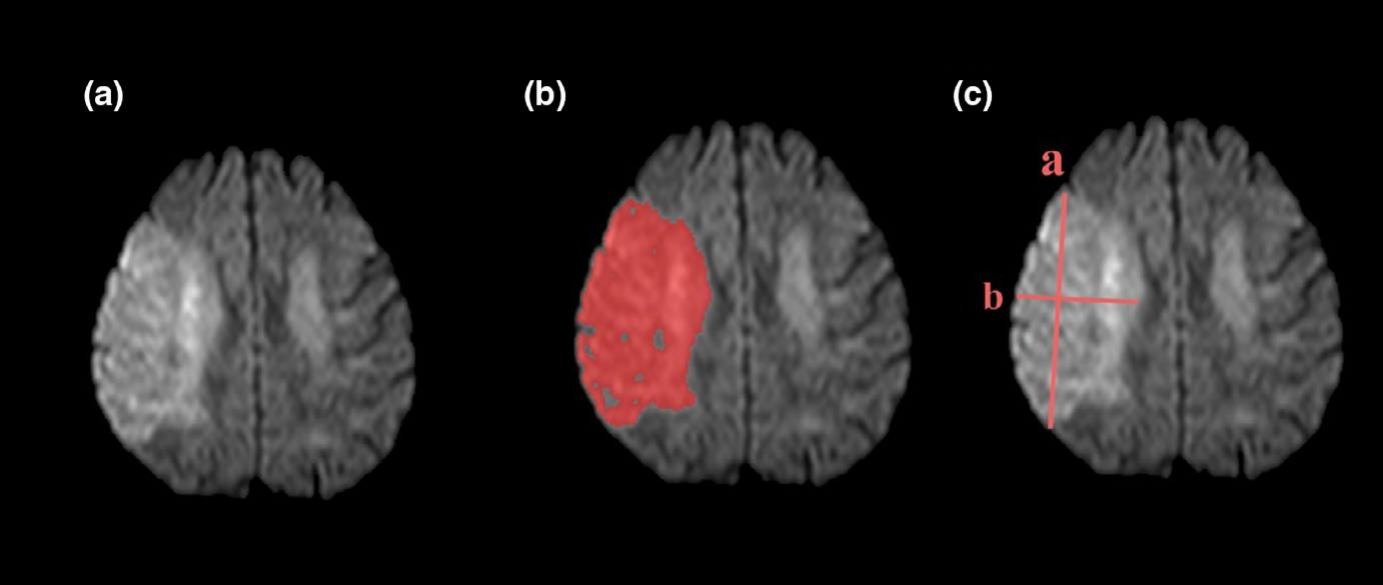
(A) Example patient with a right‐sided middle cerebral artery (MCA) stroke visible on diffusion‐weighted imaging (DWI) at baseline. (B) Based on manual delineation of the acute lesion, lesion volume was estimated to be 154.9 ml. (C) The largest diameter (*a* = 9.2 cm) and the corresponding largest orthogonal diameter (*b* = 4.8 cm) resulted in an od‐value of 44.2 (i.e., correct estimation of lesion size > 100 ml). Calculation of ABC/2 = (9.2 × 4.8) × 13 slices × 0.25 cm slice thickness resulted in an estimated volume of 143.5 ml (i.e., correct estimation of lesion size > 100 ml)

In order to assess lesion volume applying both techniques, for each patient the DWI image demonstrating the largest lesion diameter was selected. The maximum lesion diameter (*a*) was drawn on the chosen slice, and subsequently, a second maximal orthogonal (or perpendicular) diameter (*b*) was measured on the same DWI slice. Multiplication of both values (*a × b*) led to the od‐value (Figure 1). Based on a previous study, od‐value cutoffs of 32 and 42 were applied for estimation of lesion size >70 ml and >100 ml, respectively (Fiebach et al., 2015).

The ABC/2 method requires additional multiplication of the od‐value by amount of slices in which lesion was visible multiplied by the slice thickness (range 2.5–5 mm); this provides a direct estimate of lesion size in milliliters. In order to achieve actual volumes from od‐value measurements, the following formula was applied based on previously published studies (Fiebach et al., 2015; Lansberg et al., 2007):

$$\mathrm{Volume}\mathrm{in}\mathrm{ml}=1.1\left(a\times b\right)+0.03{\left(a\times b\right)}^{2}$$

### 2.4 Statistical analysis

Both methods were then compared to manually delineated volumes for estimation of lesion sizes of >70 ml and >100 ml. Statistical analysis was performed using SPSS (version 16). Sensitivity, specificity, and C statistic for predicting infarct sizes >70 ml and >100 ml were determined for both methods: od‐value and ABC/2. Percent overestimation of median volume was calculated for each method by comparing medians (Pedraza et al., 2012).

## 3 RESULTS

In the AXIS2 trial, 328 patients were enrolled; 90 patients were excluded from analysis due to movement artifacts (*n* = 3) and incompatibility of DWI raw data with our image viewing software (MRICroN and Efilm; *n* = 87). Basic demographics and baseline clinical parameters are summarized in Table 1. Manual delineation of lesion volume showed infarct volumes of >70 ml in 49 patients (20.6%) and >100 ml in 37 patients (15.5%).

**TABLE 1:** Basic demographics and baseline clinical parameters of study group

Basic demographics	
*N*	238
Age, median (Interquartile range [IQR])	71 (63–77)
Female, % (*n*)	47.5% (113)
BMI, median (IQR)	26.8 (24.5–30.1)
NIHSS on admission, median (IQR)	11 (9–17)
Manual delineation lesion volume in ml, median (IQR)	26 (10.7–62.2)
Number of slices with DWI restriction, median (IQR)	8 (6–11)

C statistics were 0.85 for od‐value and 0.87 for ABC/2. Assessment of lesion volume using od‐value and ABC/2 was tested with respect to accurate identification of lesions >70 ml and >100 ml (Table 2). ABC/2 led to an overestimation of median lesion volume of 29.9%.

**TABLE 2:** Comparison of the ABC/2 method and od‐value in terms of predicting lesion sizes of >70 ml and >100 ml

	Sensitivity (CI)	Specificity (CI)	Accuracy (CI)	PPV (CI)	NPV (CI)
Lesion size > 70 ml					
Od‐value 32	87.8 (75.2–95.4)	93.1 (88.5–96.3)	92.0 (0.88–0.95)	76.8 (63.6–87.0)	96.7 (93.0–98.8)
ABC/2	93.9 (83.1–98.7)	85.7 (79.9–90.4)	87.4 (0.83–0.92)	63.0 (50.9–74.0)	98.2 (94.8–99.6)
Lesion size > 100 ml					
Od‐value 42	70.3 (53.0–84.1)	97.0 (93.6–98.9)	92.9 (0.90–96)	81.3 (63.6–92.8)	94.7 (90.6–97.3)
ABC/2	94.6 (81.6–99.3)	93.0 (88.6–96.1)	93.3 (0.90−0.97)	71.4 (56.7–83.4)	98.9 (96.2–99.9)

## 4 DISCUSSION

While several trials have already used prespecified thresholds for patient recruitment based on lesion size (Albers et al., 2006; Lansberg et al., 2012), the reliability of applied thresholds remains debatable. This is the first study to investigate the specificity, sensitivity, and accuracy of two new diameter‐based methods for the estimation of lesion size in a large, multicenter database. Two orthogonal maximum lesion diameter methods—a 2‐axis od‐value cutoff and a 3‐axis ABC/2 calculation—were compared to volumetric measurements of lesion size. While a previous study found od‐value to have superior accuracy to ABC/2 (Fiebach et al., 2015), we found that ABC/2 and od‐value perform similarly in estimating lesion size in terms of C statistic and accuracy (Table 2).

While the sensitivities of the techniques are similar, ABC/2 tends to systematically overestimate lesion volume by up to 30%, leading to a lower specificity compared to od‐value (Table 2). These results stand in agreement with previous data reported on the ABC/2 technique (Fiebach et al., 2015; Pedraza et al., 2012). Low sensitivity may lead to false inclusion of patients with large infarction and therefore weaken interventional trials, as chances of reaching a favorable outcome in these patients are less likely. On the other hand, poor specificity due to overestimation may not only cause delayed recruitment but, more importantly, exclude patients who are likely to benefit from treatment. Therefore, a high specificity of lesion‐estimation tools is essential.

Approximately 25% of patients from the AXIS2 database were excluded due to an incompatibility of raw DWI data with our local viewing software. However, we found no significant differences in terms of basic demographics and baseline clinical parameters between included and excluded patients. Of note, AXIS2 patients were screened within 9 hr; therefore, translation of these results to other patient groups should be viewed with caution.

Nevertheless, this is the first large, multicenter study to show that these two simple diameter‐based methods are reliable and accurate tools for the rapid assessment of lesion size. While both methods perform similarly in terms of accuracy, ABC/2 tends to overestimate lesion size resulting in a lower specificity. Nonetheless, both methods may enable accurate patient recruitment with prespecified maximum infarct volumes. In the time‐sensitive setting of acute stroke, diameter‐based infarct estimation is a reliable tool for harmonization of a study cohort and is preferable to manual delineation.

## CONFLICTS OF INTEREST

CN: consulting, lecture fees, travel grants from Bayer, Boehringer Ingelheim, Bristol‐Myers Squibb, Pfizer, Sanofi, Aventis. JBF: consulting, lecture, advisory board fees from Abbvie, BioClinica, Biogen, BS, Brainomix, Cerevast, Daiich‐Sankyo, EISAI, Eli Lilly, Guerbet, Merck, Novartis, TauRx. ME reports grants from Bayer and fees paid to the Charité from Bayer, Boehringer Ingelheim, BMS/Pfizer, Daiichi Sankyo, Amgen, GSK, Sanofi, Covidien, and Novartis, all outside of the submitted work.

## AUTHOR CONTRIBUTIONS

AK involved in conceptualization and design project, data analysis (manual delineation of lesions, application of diameter‐based methods), statistical analysis, and drafting of manuscript. JS analyzed the data (delineation of lesions, application of diameter‐based methods) and involved in statistical analysis and critical appraisal of manuscript. BS conceptualized the project, involved in statistical analysis, interpreted the data, and involved in critical appraisal of the manuscript. CN and ME conceptualized the project, interpreted the data, and involved in critical appraisal of manuscript. JBF involved in conceptualization and design of project, acquisition of data, statistical analysis, interpretation of data, critical appraisal of manuscript.

### Peer Review

The peer review history for this article is available at https://publons.com/publon/10.1002/brb3.1828.

## Data Availability

The data that support the findings of this study are available from the corresponding author upon reasonable request. The data are not publicly available as they contain information that could compromise patient privacy.
